# Chicago Medical College

**Published:** 1876-03

**Authors:** N. S. Davis


### Clinics.

### CHICAGO MEDICAL COLLEGE.

*Medical Clinic by* Prof. N. S. Davis, *February* 5, 1876.

### HEPATIC DISEASE, WITH JAUNDICE.

Case I. A laboring man, about 30 years old, first presented himself at the clinic one week since, when his history was briefly stated as follows: About two years before, while actively engaged in physical exercise, he drank freely of cold water, and was soon attacked with severe pain in the epigastrium, which continued for hours, and was followed by some tenderness for several days. Similar attacks of pain recurred at intervals of a few weeks, generally coming on suddenly, and after a few hours, ceasing with equal suddenness, but leaving a more permanent sense of soreness in the epigastrium and some derangement of digestion, until six or eight months had passed. The severe paroxysms of pain then ceased, and the patient gradually became jaundiced, with a constant feeling of heaviness in the epigastric region, increased by taking food, and accompanied by impairment of appetite, general feeling of weakness, and a moderate loss of flesh. When he presented himself at the clinic last week, all these symptoms were present in a marked degree. The skin and eyes were colored deep yellow; the urine was rather scanty, and of a dark, yellowish brown color; tongue furred; pulse soft, but natural in frequency; temperature natural; bowels loose, to the extent of three or four passages a day, with some tenesmus, and more or less gaseous eructations after taking food. The stools were white or clay colored. For several months past, the patient had been much annoyed, especially during the nights, by a papular eruption upon the skin, accompanied by severe itching. A physical examination of the chest and abdomen revealed no positive signs of disease except in the epigastrium. There was no apparent enlargement of either hypochondriac region, and only the natural area of dullness over the right lobe of the liver. But directly over the sulcus dividing the left from the right lobe of this organ, a hard body was felt projecting downward two inches below the margin of the ribs, and extending entirely across the epigastrium, becoming lost under the margin of the ribs of the left side. The lower thin margin of this swelling could be easily traced with the fingers, and by percussion. Its size, shape, and position, were sufficient to identify it as an enlarged condition of the left lobe of the liver.

From the history of the case and the existing symptoms, the lecturer inferred that the early attacks of sudden and severe pain in the upper part of the abdomen, were probably caused by the passage of biliary calculi through the hepatic duct, accompanied and followed by some degree of inflammation in the membrane lining the ducts, and extending into the left lobe of the liver. Such inflammation continuing in a chronic form would induce sufficient thickening of the membrane to cause the continuous obstruction to the flow of the bile, and consequently the protracted jaundice, with slow enlargement of the diseased portion of the liver. This view of the case was strengthened by the manifest irritation of the mucous membrane of the intestines, as indicated by the colicky pains and moderate diarrhœa, as well as by the heavy, sick feeling after taking any kind of food.

The lecturer stated that impaction of the calculi in the gall bladder, too large to enter into the ducts, sometimes led to long continued jaundice and more or less distress in the epigastrium. In such cases, however, the stools seldom become entirely white or clay-colored, because some bile will continue to discharge through that part of the duct not directly connected with the gall-bladder ; and the latter can generally, in lean subjects, be felt with the fingers projecting below the margin of the liver at the sulcus. This is not the case here. The principal question of doubt admitted by the lecturer in this case was, whether the enlargement of the left lobe of the liver was owing to the chronic inflammation or to cancerous or tubercular disease.

The duration of the patient's sickness, with the absence of any well marked general emaciation or special cachexia, strongly countenanced the opinion that it was of inflammatory origin. The existing diarrhœa and griping pains may have been induced by the too free use of drastic cathartics which are often resorted to in such cases with the idea of arousing the action of the liver,—a practice characterized by the lecturer as very injurious, and resulting from the failure to distinguish between deficient hepatic secretion and obstructions in the hepatic duct preventing the secretion from escaping through its natural channel. As there was in the existing aspect of the case, plain evidences of chronic inflammation in the mucous membrane, both of the intestines and hepatic ducts, with a probable excess of cholesterine in the bile, the first object of treatment should be to remove these morbid conditions. For improving the mucous membranes the patient was directed to take a teaspoonful of the following solution before each meal and at bed time: ℞. Acid. Carbolic, grs. viij; Glycerinæ pur., ʒ ss.; Tinct. Gelsemin., ℥ ss.; Camph. Tinct. Opii, ʒ ij; Aquæ, ℥ ij. Mix.

To change the quality of the bile he was to take five minims of nitric acid, in a wine-glass full of sweetened water after each meal. The diet was to be restricted mostly to milk and farinaceous articles.

He has now been following this treatment one week, and his stomach is less nauseated; bowels move only once a day, passages consistent, and the patient says, slightly yellow. There is less tenderness over the epigastrium, and more appetite. In all other respects the symptoms remain the same as above. The lecturer remarked that the same treatment should continue another week. If during that time the mucous membranes of the stomach and intestines remained free from irritation, and the enlargement of the left lobe of the liver remained unchanged, something with greater alterative effect should be substituted in the place of the carbolic acid mixture.

The following was suggested as one of the most efficient alteratives for reducing chronic enlargement of the liver, whenever it was well borne by the stomach : ℞. Ammon. Hydrochlor., ʒ iij ; Hydrarg. Bichlorid, gr. j ; Conii Fl. Ext., ʒ iv ; Glycyrrhizæ Syr. ℥ iij ss. Mix. A teaspoonful three or four times daily.

---

A Simple Test for Ascertaining the Presence of Blood in Liquids or in Cloth.—For physicians and clinical instructors, the following method of discovering the presence of blood may be useful, especially in the examination of urine ; it combines simplicity with absolute certainty : Mix in a test-tube two cubic centimetres of *tincture of guaiac* with the equal volume of *oil of turpentine*, and then add a few drops of the urine which is to be examined. If it contains any blood, even in minutest quantity, the whole mixture at once shows a more or less intensely blue color, sometimes a deep indigo, while this coloration is produced neither by normal urine nor by urine containing albumen or pus. If you wish to ascertain whether stains in linen, wood, etc., contain blood, you proceed this way : Dissolve five grammes of guaiac in 100 cubic centim. of absolute alcohol and filter the solution ; then mix five cubic centim. of this solution with the same volume of rectified oil of turpentine, and put into this mixture the small piece of linen, wood, etc., the suspicious stains of which were previously treated with warm diluted acetic acid. The presence of blood will at once show itself by a blue color.

